# Supplementary material for: Cell and molecular transitions during efficient dedifferentiation
Source: eLife. 2020 Apr 7;9:e55435. doi: 10.7554/eLife.55435 (PMC7190356; doi:10.7554/eLife.55435)
Supplement: Supplementary file 1. [file elife-55435-supp1.docx]

| **Key Resources Table** | | | | |
| --- | --- | --- | --- | --- |
| **Reagent type (species) or resource** | **Designation** | **Source or reference** | **Identifiers** | **Additional information** |
| strain, strain background (*Dictyostelium*) | AX2 | MRC LMB | AX2, wild-type | Cell line supplied by Kay lab |
| strain, strain background (*Dictyostelium*) | DH1 | Chubb et al. 2000 | DH1, wild-type |  |
| strain, strain background (*Dictyostelium*) | AX4 | Sawai et al. 2007 | AX4, wild-type |  |
| cell line (*Dictyostelium*) | *bzpS* | This paper |  | Knock-out mutant in AX2 |
| cell line (*Dictyostelium*) | *mybD* | This paper |  | Knock-out mutant in AX2 |
| cell line (*Dictyostelium*) | *nfyA* | This paper |  | Knock-out mutant in AX2 |
| cell line (*Dictyostelium*) | *DDB_G0269374* | This paper |  | Knock-out mutant in AX2 |
| cell line (*Dictyostelium*) | *DDB_G0272386* | This paper |  | Knock-out mutant in AX2 |
| cell line (*Dictyostelium*) | *DDB_G0281091* | This paper |  | Knock-out mutant in AX2 |
| cell line (*Dictyostelium*) | *bzpI* | This paper |  | Knock-out mutant in AX2 |
| cell line (*Dictyostelium*) | *eriA* | This paper |  | Knock-out mutant in AX2 |
| cell line (*Dictyostelium*) | *fslN* | This paper |  | Knock-out mutant in AX2 |
| cell line (*Dictyostelium*) | *gbpD* | This paper |  | Knock-out mutant in AX2 |
| cell line (*Dictyostelium*) | *jcdA* | This paper |  | Knock-out mutant in AX2 |
| cell line (*Dictyostelium*) | *nfaA* | This paper |  | Knock-out mutant in AX2 |
| cell line (*Dictyostelium*) | *ptpB* | This paper |  | Knock-out mutant in AX2 |
| cell line (*Dictyostelium*) | *DDB_G0277531* | This paper |  | Knock-out mutant in AX2 |
| cell line (*Dictyostelium*) | *ctnB* | This paper |  | Knock-out mutant in AX2 |
| cell line (*Dictyostelium*) | *gefAA* | This paper |  | Knock-out mutant in AX2 |
| cell line (*Dictyostelium*) | *gefS* | Williams et al. 2019 |  | Knock-out mutant in AX2 |
| cell line (*Dictyostelium*) | *gtaN* | This paper |  | Knock-out mutant in AX2 |
| cell line (*Dictyostelium*) | *krsB* | Williams et al. 2019 |  | Knock-out mutant in AX2 |
| cell line (*Dictyostelium*) | *omt5* | This paper |  | Knock-out mutant in AX2 |
| cell line (*Dictyostelium*) | *pakE* | Sawai et al. 2007 |  | Knock-out mutant in AX4 |
| cell line (*Dictyostelium*) | *rasG* | Veltman et al. 2016 |  | Knock-out mutant in AX2 |
| cell line (*Dictyostelium*) | *sigB* | This paper |  | Knock-out mutant in AX2 |
| cell line (*Dictyostelium*) | *sodC* | This paper |  | Knock-out mutant in AX2 |
| cell line (*Dictyostelium*) | *tagA* | This paper |  | Knock-out mutant in AX2 |
| cell line (*Dictyostelium*) | *xacB* | This paper |  | Knock-out mutant in AX2 |
| cell line (*Dictyostelium*) | *zakA* | This paper |  | Knock-out mutant in AX2 |
| cell line (*Dictyostelium*) | *DDB_G0268696* | This paper |  | Knock-out mutant in AX2 |
| cell line (*Dictyostelium*) | *DDB_G0269040* | This paper |  | Knock-out mutant in AX2 |
| cell line (*Dictyostelium*) | *DDB_G0270436* | This paper |  | Knock-out mutant in AX2 |
| cell line (*Dictyostelium*) | *DDB_G0270480* | This paper |  | Knock-out mutant in AX2 |
| cell line (*Dictyostelium*) | *DDB_G0272364* | This paper |  | Knock-out mutant in AX2 |
| cell line (*Dictyostelium*) | *DDB_G0272434* | This paper |  | Knock-out mutant in AX2 |
| cell line (*Dictyostelium*) | *DDB_G0274177* | This paper |  | Knock-out mutant in AX2 |
| cell line (*Dictyostelium*) | *DDB_G0275621* | This paper |  | Knock-out mutant in AX2 |
| cell line (*Dictyostelium*) | *DDB_G0276549* | This paper |  | Knock-out mutant in AX2 |
| cell line (*Dictyostelium*) | *DDB_G0278193* | This paper |  | Knock-out mutant in AX2 |
| cell line (*Dictyostelium*) | *DDB_G0279851* | This paper |  | Knock-out mutant in AX2 |
| cell line (*Dictyostelium*) | *DDB_G0280067* | This paper |  | Knock-out mutant in AX2 |
| cell line (*Dictyostelium*) | *DDB_G0283057* | This paper |  | Knock-out mutant in AX2 |
| cell line (*Dictyostelium*) | *DDB_G0288203* | This paper |  | Knock-out mutant in AX2 |
| cell line (*Dictyostelium*) | *DDB_G0289907* | This paper |  | Knock-out mutant in AX2 |
| cell line (*Dictyostelium*) | *DDB_G0292302* | This paper |  | Knock-out mutant in AX2 |
| cell line (*Dictyostelium*) | *DDB_G0293078* | This paper |  | Knock-out mutant in AX2 |
| cell line (*Dictyostelium*) | *DDB_G0293562* | This paper |  | Knock-out mutant in AX2 |
| cell line (*Dictyostelium*) | *forG* | This paper |  | Knock-out mutant in AX2 |
| cell line (*Dictyostelium*) | *rasS* | Chubb et al. 2000 |  | Knock-out mutant in DH1 |
| cell line (*Dictyostelium*) | CryS-mNeonGreen | Antolovic et al. 2019 |  | Knock-in tag in AX3 |
| cell line (*Dictyostelium*) | Act8-mNeonGreen | Tunnacliffe et al 2018 |  | Knock-in tag in AX3 |
| transfected construct (*Dictyostelium*) | A15-mCherry-PCNA | Miermont et at. 2019 |  |  |
| antibody | Anti-pAMPK alpha Thr 172, (rabbit monoclonal) | Cell Signalling Technology | CST#2535 | 1/500 |
| antibody | Anti-p4E-BP1 Thr 37/46 (rabbit polyclonal) | Cell Signalling Technology | CST#9459 | 1/500 |
| antibody | Anti-Histone H3  (rabbit polyclonal) | Abcam | #ab1791 | 1/1000 |
| antibody | Anti-rabbit IgG HRP-linked (Donkey whole antibody) | GE healthcare | #NA934 | pAMPK: 1/10000  p4E-BP1: 1/5000  H3: 1/10000 |
| commercial assay or kit | NEBNext mRNA isolation kit | New England Biolabs | #E7490L |  |
| commercial assay or kit | NEBNext Ultra Directional RNA Library preparation kit | New England Biolabs | #E7420 |  |
| commercial assay or kit | NEBNext Ultra II Directional RNA Library preparation kit | New England Biolabs | #E7760 |  |
| commercial assay or kit | Mid-output 150-cycle kit | Illumina | 20024904 |  |
| commercial assay or kit | NextSeq®500 v2 High-output 150-cycle kit | Illumina | 20024907 |  |
| commercial assay or kit | Chromium™ 3’ Library & Gel Bead Kit v2 | 10x Genomics | PN-120267 |  |
| software, algorithm | Cell Ranger v2 | 10x Genomics | RRID:SCR_017344 |  |
| software, algorithm | FastQC | FastQC | RRID:SCR_014583 |  |
| software, algorithm | Tophat | University of California; Berkeley; University of Maryland | RRID:SCR_013035 |  |
| software, algorithm | HTSeq | EMBL | RRID:SCR_005514 |  |
| software, algorithm | Integrative Genomics Viewer (IGV) | Broad Institute | RRID:SCR_011793 |  |
| software, algorithm | R Project for Statistical Computing | R Project for Statistical Computing | RRID:SCR_001905 |  |
| software, algorithm | Wolfram Mathematica | Wolfram | RRID:SCR_014448 |  |
| software, algorithm | MATLAB | MathWorks | RRID:SCR_001622 |  |
