# Supplementary material for: Cell and molecular transitions during efficient dedifferentiation
Source: eLife. 2020 Apr 7;9:e55435. doi: 10.7554/eLife.55435 (PMC7190356; doi:10.7554/eLife.55435)
Supplement: Transparent reporting form [file elife-55435-transrepform.docx]

***eLife’s* transparent reporting form**

We encourage authors to provide detailed information *within their submission* to facilitate the interpretation and replication of experiments. Authors can upload supporting documentation to indicate the use of appropriate reporting guidelines for health-related research (see [EQUATOR Network](http://www.equator-network.org/%20)), life science research (see the [BioSharing Information Resource](https://biosharing.org/)), or the [ARRIVE guidelines](http://www.plosbiology.org/article/info:doi/10.1371/journal.pbio.1000412) for reporting work involving animal research. Where applicable, authors should refer to any relevant reporting standards documents in this form.

If you have any questions, please consult our Journal Policies and/or contact us: [editorial@elifesciences.org](mailto:editorial@elifesciences.org).

**Sample-size estimation**

- You should state whether an appropriate sample size was computed when the study was being designed
- You should state the statistical method of sample size computation and any required assumptions
- If no explicit power analysis was used, you should describe how you decided what sample (replicate) size (number) to use

Please outline where this information can be found within the submission (e.g., sections or figure legends), or explain why this information doesn’t apply to your submission:

Population transcriptomics: two replicates for each condition (4 in total, over 2 dedifferentiation conditions), individual genes were not followed up unless reproduced between the two conditions. Results were cross-referenced to single cell transcriptomic and Northern blot data on independent cell preparations. Screening transcriptomics on TF mutants was carried out with 1 replicate. No strong effects were observed. Transcriptomics on *forG* mutants was carried out twice.

Single cell transcriptomics: two biological replicates, results also cross-referenced to population RNAseq and Northern data to ascertain reproducibility.

Imaging: 4 replicates carried out for gene expression versus motility/division time. Data were not analysed until all had been collected. For cell fate versus motility/division time, 2 replicates were carried out, without an effect. Data were not analysed until all had been collected

Genetics: Each cell line was initially tested once. If an effect was initially observed, testing was repeated. Effects reported for *forG* and *rasS* mutants were repeated, by Northern blot, at least 3 times.

**Replicates**

- You should report how often each experiment was performed
- You should include a definition of biological versus technical replication
- The data obtained should be provided and sufficient information should be provided to indicate the number of independent biological and/or technical replicates
- If you encountered any outliers, you should describe how these were handled
- Criteria for exclusion/inclusion of data should be clearly stated
- High-throughput sequence data should be uploaded before submission, with a private link for reviewers provided (these are available from both GEO and ArrayExpress)

Please outline where this information can be found within the submission (e.g., sections or figure legends), or explain why this information doesn’t apply to your submission:

All replicate sizes reported in the legends. We define experiments as “independent”- this means a biological, not a technical replicate.

Consideration of outliers was only applied to scRNAseq data, using standard approaches to remove cells with extreme sequencing depths and those lacking contiguous sections of the genome. These treatments are described in the SI.

Sequencing upload to GEO is in process. Data can be accessed from the UCL data repository at [https://figshare.com/s/82af36190af4ceb41da0](https://eur01.safelinks.protection.outlook.com/?url=https%3A%2F%2Ffigshare.com%2Fs%2F82af36190af4ceb41da0&data=02%7C01%7C%7C83c88bb6a7fa4294d1fa08d7aa307502%7C1faf88fea9984c5b93c9210a11d9a5c2%7C0%7C0%7C637164997400077345&sdata=JxBG8vtIlhm59eLHsnKr4XRup0%2FeRAy%2FSz7Kaeb9sFA%3D&reserved=0).

**Statistical reporting**

- Statistical analysis methods should be described and justified
- Raw data should be presented in figures whenever informative to do so (typically when N per group is less than 10)
- For each experiment, you should identify the statistical tests used, exact values of N, definitions of center, methods of multiple test correction, and dispersion and precision measures (e.g., mean, median, SD, SEM, confidence intervals; and, for the major substantive results, a measure of effect size (e.g., Pearson's r, Cohen's d)
- Report exact p-values wherever possible alongside the summary statistics and 95% confidence intervals. These should be reported for all key questions and not only when the p-value is less than 0.05.

Please outline where this information can be found within the submission (e.g., sections or figure legends), or explain why this information doesn’t apply to your submission:

Tests and p-values are stated in figure legends. We used Mann-Whitney, chi-squared and KS tests. Mann-Whitney tests were chosen to avoid any assumptions on the nature of the data. Chi-squared tests were used to compare categories with a hypothetical ratio. KS tests were used to compare distributions without making any assumptions about the data.

In general the nature of the average, sample sizes, correlation values (if appropriate), statistical tests and p-values, measures of dispersion (usually SD) are shown in the figures and/or reported in the figure legends. Exact p-values are reported where provided by GraphPad Prism software, and for all non-significant effects.

(For large datasets, or papers with a very large number of statistical tests, you may upload a single table file with tests, Ns, etc., with reference to sections in the manuscript.)

**Group allocation**

- Indicate how samples were allocated into experimental groups (in the case of clinical studies, please specify allocation to treatment method); if randomization was used, please also state if restricted randomization was applied
- Indicate if masking was used during group allocation, data collection and/or data analysis

Please outline where this information can be found within the submission (e.g., sections or figure legends), or explain why this information doesn’t apply to your submission:

Allocation for clusters for gene expression changes (based on population RNAseq) was based upon unbiased hierarchical clustering, as described in the supplementary methods. Group allocation for imaging experiments emerged from the data, either via unsupervised clustering, or by comparing different features of the data collected on the same cell population.

**Additional data files (“source data”)**

- We encourage you to upload relevant additional data files, such as numerical data that are represented as a graph in a figure, or as a summary table
- Where provided, these should be in the most useful format, and they can be uploaded as “Source data” files linked to a main figure or table
- Include model definition files including the full list of parameters used
- Include code used for data analysis (e.g., R, MatLab)
- Avoid stating that data files are “available upon request”

Please indicate the figures or tables for which source data files have been provided:

Sequencing data can be accessed as described above.

The numerical data that forms the basis of the graphs in the study has been included in the submission as Excel Tables, labeled according to the relevant figures.
